# Toward High-Performance Li Metal Anode via Difunctional Protecting Layer

**DOI:** 10.3389/fchem.2019.00572

**Published:** 2019-08-20

**Authors:** Jinlei Gu, Chao Shen, Zhao Fang, Juan Yu, Yong Zheng, Zhanyuan Tian, Le Shao, Xin Li, Keyu Xie

**Affiliations:** ^1^State Key Laboratory of Solidification Processing, Center for Nano Energy Materials, School of Materials Science and Engineering, Northwestern Polytechnical University and Shaanxi Joint Laboratory of Graphene (NPU), Xi'an, China; ^2^School of Metallurgical Engineering, Xi'an University of Architecture and Technology, Xi'an, China; ^3^Shaanxi Coal and Chemical Technology Institute Co., Ltd, Xi'an, China

**Keywords:** AgNO_3_, difunctional protecting layer, Li anode, Li-S battery, Li dendrites

## Abstract

Li-metal batteries are the preferred candidates for the next-generation energy storage, due to the lowest electrode potential and high capacity of Li anode. However, the dangerous Li dendrites and serious interface reaction hinder its practical application. In this work, we construct a difunctional protecting layer on the surface of the Li anode (the AgNO_3_-modified Li anode, AMLA) for Li-S batteries. This stable protecting layer can hinder the corrosion reaction with intermediate polysulfides (Li_2_S_x_, 4 ≤ x ≤ 8) and suppress the Li dendrites by regulating Li metal nucleation and depositing Li under the layer uniformly. The AMLA can cycle more than 50 h at 5 mA cm^−2^ with the steady overpotential of lower than 0.2 V and show high capacity of 666.7 mAh g^−1^ even after 500 cycles at 0.8375 mA cm^−2^ in Li-S cell. This work makes great contribution to the protection of the Li anode and further promotes the practical application.

## Introduction

Li metal is the most promising anode material for the next-generation batteries (Li-metal batteries), such as Li-S batteries (Su et al., 2018) and Li-air batteries (Cao et al., 2019), due to the lowest electrode potential (−3.04 V, compare to the hydrogen electrode) and the high capacity (3,860 mAh g^−1^, which is 10 times than the commercial graphite) (Liu et al., 2016; Yan K. et al., 2016; Liang et al., 2017; Zhang K. et al., 2017; Bai et al., 2018; Cheng et al., 2018, 2019; Li et al., 2018; Terlicka et al., 2019). However, the intense chemical activity of Li metal leads to sever interface reactions between Li and electrolyte, which results in low Coulomb efficiency and increasing interface impedance (Zheng et al., 2014; Shen et al., 2018). Besides, the inhomogeneous Li deposition leads to forming the dangerous Li dendrites on the Li anode (Tao et al., 2017). During the battery cycling, the Solid Electrolyte Interface (the SEI film) to rupture and re-repair continuously since the uncontrollable Li dendrites. It will trigger the serious side reaction to consume the electrolyte and Li (Hou et al., 2019). More severely, the Li dendrites will pierce through the separator, and cause serious safety hazard (the battery short-circuited, producing a large amount of joule heat and triggering an explosion; Peng et al., 2016; Yang et al., 2017; Zhang et al., 2018; Hou et al., 2019). Thus, the Li anode cannot be commercialized without addressing the above problems (Cheng et al., 2018).

Recently, a variety of approaches are employed to hinder the interface reaction and suppress the Li dendrites to promote the practicality of the Li anode. Firstly, the electrolyte additives are added into electrolyte to increase the stability and dense of the SEI film to suppress the serious interface reaction (Zhang, 2012; Jing et al., 2015; Xie et al., 2016; Shiga et al., 2017; An et al., 2019; Chen et al., 2019; Wang et al., in press). However, the SEI film still has limited structure strength, the growth of Li dendrites will destroy it during the cycles. In addition, researchers suppress the Li dendrites through controlling nucleation sites of Li (Zhang et al., 2016, 2018; Jin et al., 2017; Pei et al., 2017; Zhang R. et al., 2017; Hou et al., 2019; Li et al., 2019) and forming a protective layer (Hiratani et al., 1988; Choi et al., 2004; Kozen et al., 2015; Liu et al., 2016, 2018; Peng et al., 2016; Liang et al., 2017; Xie et al., 2017; Bai et al., 2018; Li et al., 2018; Wang et al., 2019) on the Li anode. For example, Liang et al. form a Li-rich composite alloy/LiCl layer on the Li anode (Liang et al., 2017). The Li^+^ goes through the protecting layer fast to let Li be deposited under it to suppress the Li dendrites because of the high ionic conduction of the alloy layer. Nevertheless, the uneven deposition of Li is not improved, which means that Li dendrites still are formed. Moreover, Yang et al. struct a 3D host material with ubiquitous and uniform nanoseeds (the ultrafine Ag nanoparticles) to regulate the Li nucleation and deposition homogeneously (Yang et al., 2017). However, this method increases the interface area between electrolyte and Li anode to aggravate the interface reaction and cannot protect the SEI film as the large volume change during the deposition and dissolution process of the Li. Thurs, single method cannot protect the Li anode in the Li-metal batteries well. It is very important to form a protecting layer on the Li anode, which can lead the homogeneous deposition of Li under it and remain the stable SEI film to hinder the interface reaction.

Herein, we form such difunctional protecting layer on the Li anode as the AgNO_3_-modified Li anode (AMLA) through a simple way, dropping the solution of AgNO_3_ on the Li anode. In order to study the effect of this layer, we apply it to the Li-S batteries. This difunctional protecting layer is consisted of the LiAg alloy and the modified SEI film by LiNO_3_. On the one hand, the layer can suppress the Li dendrites to keep the integrity of the SEI film. The LiAg alloy can regulate Li nucleation and let Li depositing under the protecting layer uniformly to suppress the Li dendrites. On the other hand, the modified SEI film by LiNO_3_ can protect the Li anode from the electrolyte to suppress the corrosive reaction between Li and intermediate polysulfides (Jing et al., 2015; Li et al., 2015; Yan C. et al., 2016). The difunctional protecting layer can improve the Li anode through suppressing the Li dendrites and hindering the corrosion reaction in the Li-S batteries.

## Materials and Methods

### Synthesis of the Protecting Layer on the Li Anode

The AgNO_3_ solution used polar solvent tetrahydrofuran (THF) as the solvent to disperse the AgNO_3_. Put 50 mg AgNO_3_ (AR, ≥99.8%) in 10 ml THF (≥99.8%, HPLC) solvent in the glove box filled with argon (H_2_O < 0.1 ppm, O < 0.1 ppm). After heating and stirring for 24 h, the solution was ultrasonic disperse until uniform clarification solution is obtained. The template for making the solution is 30°C and the speed of stirring is 500 rpm min^−1^. Drop 20 μL of prepared solution onto the Li anode through using the pipette. Heat for 3 min to ensure no THF solvent residual with the template of 40°C.

### Characterizations

X-ray diffraction (XRD) was characterized by STOE PANalytical Empyrean. The experimental conditions are as follows: the radiation is the CuKα radiation and the λ is 1.5406 Å, the scanning Angle is 20–90°, the current and voltage are 40 mA and 40 kV, the scanning time is 8 min (the scanning step length is 3°, the dwell time is 20 s). X-ray photoelectron spectroscopy (XPS) characterizations were characterized by an ESCALAB250xi XPS system. Energy Dispersion Spectrum (EDS) and Scanning electron microscope (SEM) studies were carried out with FESEM, FEI Tecnai G2 F30. All of the samples were washed by the 1,2-dimethoxyethane (DME) for three times and tested after the DME evaporated.

### Electrochemical Measurements

The CR2016-type coin cells were used to study the electrochemical performance, which assembled with one 0.5 mm metallic gasket in the glove box. Celgard type 2400 polypropylene film was used as separator. The electrolyte in the experiments was that 1 M Li bis(trifluoromethane sulfonyl)imide (LiTFSI) is dissolved in the solvent consisting of 1,2-dimethoxyethane (DME) and 1,3-dioxolane (DOL), where the ratio was 1:1 by volume. There were two kinds of cells (the symmetrical cell and Li-S cell) used to study the electrochemical performance of the protecting layer. The symmetric cells used the Li anode or the AMLA on each side, as shown in the **Figure 4a**. The electrochemical performance of symmetrical cells was measured by symmetrical cycle test and electrochemical impedance spectroscopy (EIS) test. As the symmetrical cycle test, the symmetrical cells were cycled with the Li deposit amount of 0.5 mAh cm^−2^ at 1, 2, and 5 mA cm^−2^ to make the change of voltage—time curve on a LAND battery system. The frequency range of the EIS test was from 0.01 to 1,000,000 Hz and the amplitude was 10 mV, which was carried out on Solartron electrochemical workstation (1260 + 1287, England).

For the Li-S cell, the S cathode was formation: firstly, forming the slurry through mixing the S, binder (polyvinylidene fluoride, PVDF) and conductive carbon black (super-P acetylene black), at a weight ratio of 8:1:1, in the solution of N-methyl-2-pyrrolidone (NMP); then, casting the slurry on carbon-coated aluminum foil at 200 μm with the doctor blade as S cathodes; in the last, vacuum drying the cathode at 60°C for 10 h and rushing into 12 mm diameter wafer, the plate load per unit area is about 1.0 mg cm^−2^. The Li-S cell was composed of the S cathode and Li anode or the AMLA.

Galvanostatic charge-discharge tests were carried to study the electrochemical performance of the cell, and the current density was 0.5 C (1 C = 1,675 mA g^−1^) with the voltage windows of 1.7–2.8 V.

## Results and Discussion

**Figure 1:**
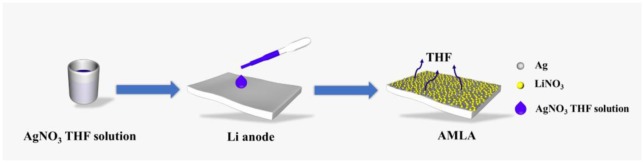
The illustration of the synthesis of the AMLA.

The AMLA is formed by a very simple way, dropping the THF solution of AgNO_3_ on the Li anode, as illustrated in Figure 1. After the redundant THF evaporates, a thin mixing layer of LiNO_3_ and Ag is formed on the Li anode and we can see the color of the Li metal changes to yellow. The mixing layer is made *in situ* spontaneous reduction of AgNO_3_ by Li, due to that the redox potential of Ag/Ag^+^ couples (0.8 V, compare to the hydrogen electrode) is much higher than the Li/Li^+^. And this reaction can be represented by the following equation:

(1) $$\begin{array}{r} {\text{AgNO}}_{3}+\text{ Li}\to \;{\text{LiNO}}_{3}\;+\text{ Ag} \end{array}$$

**Figure 2:**
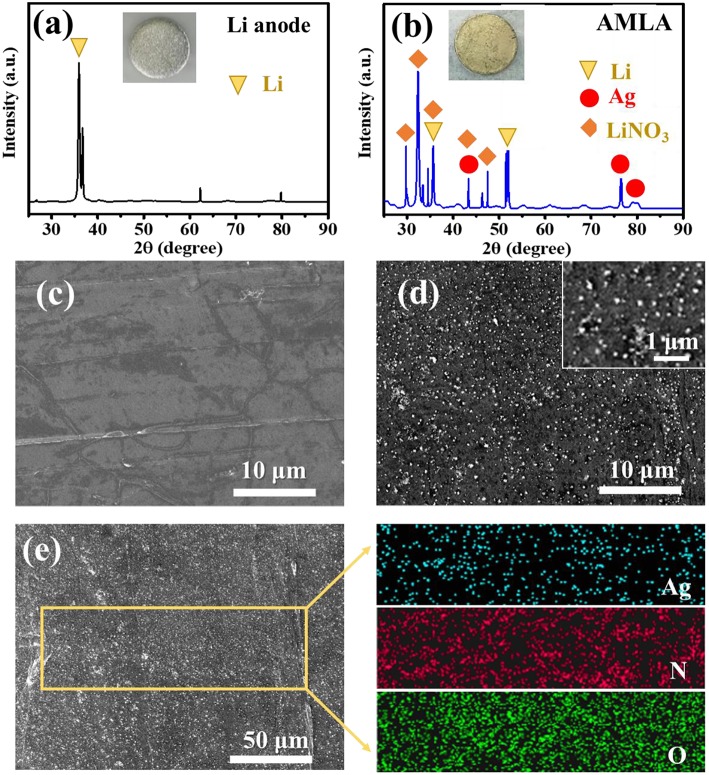
The XRD curves of Li anode **(a)** and AMLA **(b)**, and the circle sheets in the figures represent the Li anode in different states. **(c)** The SEM image of Li anode. **(d)** The SEM image of AMLA. **(e)** The EDS images of ALMA, and the blue spots represent the element Ag, the red ones represent the element N, and the green ones represent the element O.

The formation of LiNO_3_ and Ag can be confirmed through employing the XRD. The characteristic diffraction peaks of Ag (PDF#01-1167) at 44.50°, 77.63°, and 81.57° corresponding to the (200), (311), and (222) facets appear on the AMLA (Figure 2b), comparing the only characteristic diffraction peaks of Li in the Figure 2a (Taillades and Sarradin, 2004; Offor et al., 2015). In addition, there are another four characteristic diffraction peaks of LiNO_3_, which is at 24.82°, 32.10°, 35.28°, and 42.45° (Figure 2b), corresponding to the (012), (104), (006), and (113) facets in the PDF#01-1225. In addition, the morphologies of the Li anode (Figure 2c) and AMLA (Figure 2d) are characterized by the SEM. The surface of the Li anode is very smooth, while the AMLA is rough with a lot of Ag nanoparticles. The size of these nanoparticles is smaller than 100 nm and they are homogeneous distribution as confirmed by the EDS of the AMLA (Figure 2e). All of these results confirm that we form the mixing uniform layer of LiNO_3_ and Ag on the Li anode as the Equation (1).

**Figure 3:**
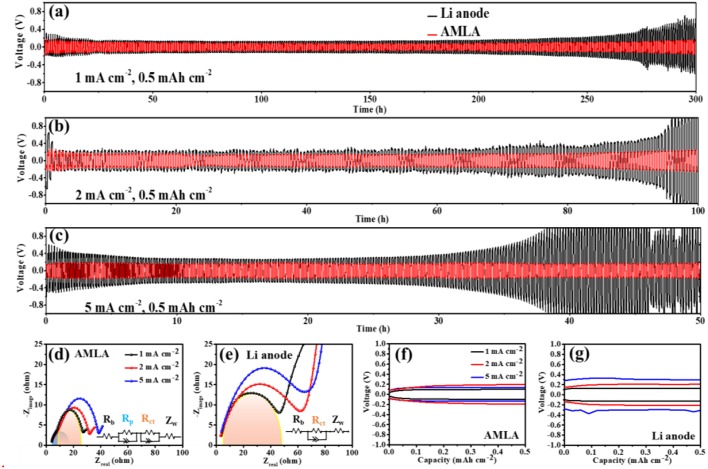
The electrochemical performances of symmetrical cells. **(a–c)** The voltage-time curves of Li anode (back) and AMLA (red) electrodes under 1, 2, and 5 mA cm^−2^current density, the deposit amount of Li^+^ is 0.5 mAh cm^−2^ for all the battery. The EIS figures of AMLA **(d)** and Li anode **(e)** after 30 times' cycles under 1 mA cm^−2^ (black), 2 mA cm^−2^ (red), and 5 mA cm^−2^ (blue) current density. The polarization voltage figures of AMLA **(f)** and Li anode **(g)** after 30 times' cycles, under 1 mA cm^−2^ (black), 2 mA cm^−2^ (red), and 5 mA cm^−2^ (blue) current density.

The electrochemical performances of the cells with the AMLA show much better due to the mixing layer of LiNO_3_ and Ag, comparing to the symmetrical cells with the Li anode (Figure 3). First of all, the symmetrical cells with the AMLA own more stable cycle performance and longer lifetime. The symmetrical cells are cycled at 1, 2, and 5 mA cm^−2^ with the Li deposit amount of 0.5 mAh cm^−2^. The voltage-time curves of the Li anode (back) and AMLA (red) are showed in the Figures 3a–c. The symmetrical cell with the Li anode is only stably cycled for 200 h with overpotential of 0.1 V, when the current density is 1 mA cm^−2^. Subsequently, the overpotential raise up with the decomposition of the electrolyte and the thicker SEI film during the cycling (Yang et al., 2017). After cycling for 300 h, the overpotential of the cell becomes higher than 0.5 V. In addition, the cell with the Li anode appears violent voltage fluctuation after 76 h at low current density of 2 mA cm^−2^ and 35 h at high current density of 5 mA cm^−2^ (Figures 3b,c), which is caused by the reaction between Li and electrolyte (Liu et al., 2018). And there is a performance that as the voltage first decreases and then increases just like a neck shape in the voltage-time curve of the symmetrical cell with the Li anode at 5 mA cm^−2^. This special curve results from the generation and growth of dendrites (Liu et al., 2016). However, the symmetrical cell with the AMLA shows much more stable cycling at different current density. As we can see, the cell with the AMLA can be cycled more than 50 h with steady overpotential, even at 5 mA cm^−2^, which is lower than 0.2 V (Figure 3c). Furthermore, the cell with the AMLA shows the lower resistance after 30 times' cycles (Figures 3d,e). The resistance is consisted of the ohmic resistance (R_b_), interfacial resistance of the electrode (R_i_), and diffusion impedance of lithium ions in solids (Z_w_) (Wu et al., 2019; Zhou et al., 2019). In the symmetrical cells, the most important resistance is the Ri, because the R_b_ (4 Ω) and the Z_w_ (the slop of the sloping line at the low frequency region are the same) are the same in different electrodes. As for the AMLA, there are two semicircles in the high frequency region of the EIS curve, due to the mixing layer of LiNO_3_ and Ag: the first one represents the lithium ion transfer impedance (R_p_) and the second one represents the charge transfer impedance (R_ct_) (Wu et al., 2019). Therefore, the R_i_ of the AMLA is consisted of R_p_ and R_ct_. However, there is only one semicircle for the Li anode, because of the two synchronous processes of lithium ion transfer and charge transfer. So, the R_i_ is the same as the R_ct_ in the Li anode. The R_i_ of the AMLA is negatively changed at different current density as 23 Ω at 1 mA cm^−2^, 26 Ω at 2 mA cm^−2^, and 34 Ω at 5 mA cm^−2^ (Figure 3d). Whereas, the R_i_ (R_ct_) of the Li anode is twice higher than the AMLA and increased with the higher current density (45 Ω at 1 mA cm^−2^, 60 Ω at 2 mA cm^−2^, and 70 Ω at 5 mA cm^−2^, Figure 3e). The hysteresis in the voltage profile is even lower than 0.2 V for the AMLA at current density of 5 mA cm^−2^, while it increased higher than 0.35 V for the Li anode (Figures 3f,g). The mixing uniform layer of LiNO_3_ and Ag can improve the electrochemistry performance in the symmetrical cells, which is formed in the AMLA before cycling.

**Figure 4:**
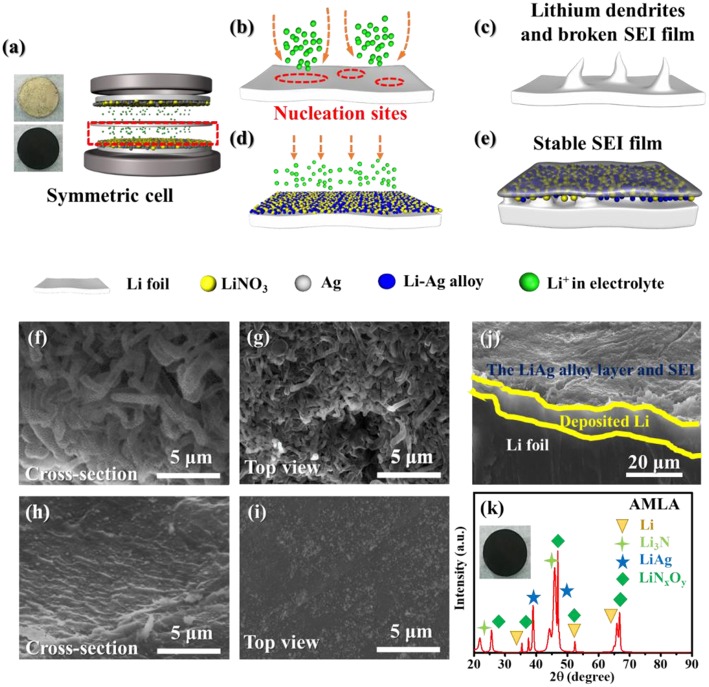
The change of anode when during charge/discharge progress and the morphology of Li anode and AMLA. **(a)** The schematic of symmetric cell. **(b,c)** The schematic of change on Li anode during charge/discharge progress. **(d,e)** The schematic of change on AMLA during charge/discharge progress. **(f,g)** SEM images of cross-section and top-view of Li anode after cycles. **(h,i)** SEM images of cross-section **(h)** and top-view **(i)** of AMLA after cycles. **(j)** SEM images of cross-section AMLA with the deposit amount of 1 mAh cm^−2^ as the current density of 1 mA cm^−2^. The XRD curves of AMLA **(k)**.

Except for the electrochemistry performance, the mixing uniform layer of LiNO_3_ and Ag changes the surface of the Li anode after cycling (Figures 4f–i). For the Li anode, there are a lot of drastic Li dendrites on the surface in the cross-section and top view SEM images (Figures 4f,g). These Li dendrites are wire shape with length of 5–10 μm and diameter of 1–2 μm. It is indicating that the nucleation and deposition of Li are inhomogeneous (Figures 4b,c). These Li dendrites will increase the interface of the Li anode contacting with electrolyte to accelerate the side reaction as violent voltage fluctuation during the cycling (Figures 3a–c). And, it will consume large amounts of electrolyte and deteriorate the cycle performance. Furthermore, these Li dendrites will pierce the separator and cause safety hazard. In contrast, the morphologies of the Li anode are much smooth without any Li dendrites (Figures 4d,e). In order to understand the mechanism of performance of the AMLA, we use the XRD to study the surface of the Li anode and AMLA anode after the cycling (Figure 4k). Once disassembling the symmetrical cell, the first thing we find that the color of the AMLA anode becomes black from yellow. It means that there are somethings formed on the anode during the cycling. In the XRD spectrum of it, there are some characteristic diffraction peaks of another three main composites except Li, corresponding to the LiAg alloy, Li_3_N, and LiN_x_O_y_, which can be represented:

(2) $$\begin{array}{rcl} \text{Ag }+\;{\text{Li}}^{+}\;+\;{\text{e}}^{-} & \to & \text{ LiAg} \end{array}$$

(3) $$\begin{array}{rcl} {\text{NO}}_{3}^{-}\;+\;{\text{Li}}^{+}+\;{\text{e}}^{-} & \to & {\text{Li}{\text{N}}_{\text{x}}\text{O}}_{\text{y}}+\text{ L}{\text{i}}_{3}\text{N} \end{array}$$

**Figure 5:**
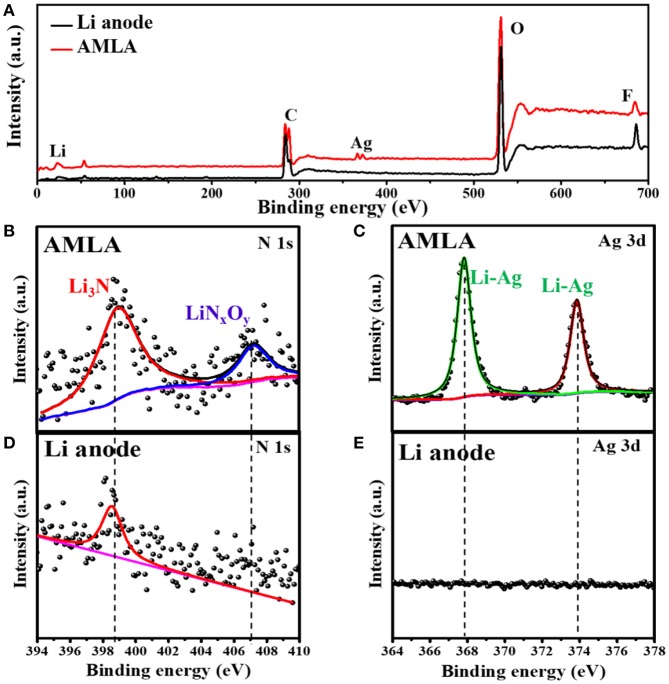
The XPS spectra of Li anode and AMLA after cycles. The full XPS spectra **(A)** of Li anode (back) and AMLA (red) electrodes after cycles. XPS spectra of different element of AMLA: **(B)** N 1s and **(C)** Ag 3d. XPS spectra of different element of Li anode: **(D)** N 1s and **(E)** Ag 3d.

**Figure 6:**
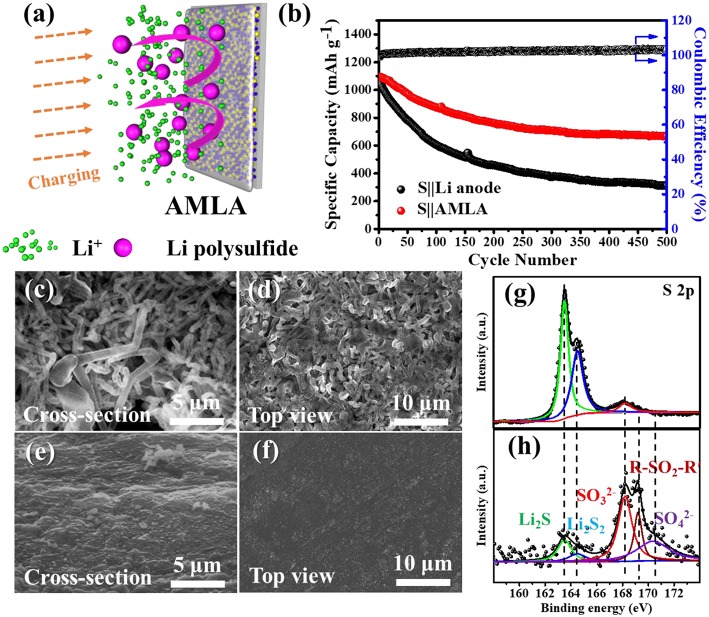
The electrochemical performances of the Li-S cells. **(a)** The schematic of great effect of AMLA in the full cell. **(b)** The cycle performances of S||AMLA and S||Li anode cells under galvanostatic test with the current density 0.5 C (1 C = 1,675 mA g^−1^). **(c,d)** SEM images of cross-section and top-view of Li anode after cycles. **(e,f)** SEM images of cross-section and top-view of AMLA after cycles. The XPS spectra of S 2p elemental: AMLA **(h)** and Li anode **(g)** electrodes after cycles.

Equation (2) is the reaction between Li and Ag to form the LiAg alloy on the electrode during cycling. Equation (3) is the decomposition of LiNO_3_ (Jing et al., 2015), which forms beyond the anode in the electrolyte. Apart from the XRD, we also use the XPS to study the surface of the Li anode and AMLA anode (Figure 5). The full XPS spectra (Figure 5A) shows: (i) the ration of Li element in the Li anode (2.6%) is lower than in the AMLA anode (10.5%); (ii) the ration of F element in the Li anode (46.0%) is higher than in the AMLA (40.8%); (iii) the peaks of Ag element come on in the AMLA anode. Without AgNO_3_, the severe interface reaction between the Li anode and electrolyte forms much by-products with F, C, O, and so on, as the higher ratio of F on the surface. In addition, these by-products thicker the SEI film, which will make less Li be detected, as the lower ratio of Li. Moreover, Ag 3d spectrum shows two peaks at binding energy of 367.84 eV for Ag 3d_5/2_ and 373.86 eV for Ag 3d_3/2_, which means the LiAg alloy is formed (Zhang et al., 2018), shown in the Figure 5C. N 1 s (Figure 5B) can be assigned to Li_3_N (398.94 eV) and LiN_x_O_y_ (407.11 eV), while no LiN_x_O_y_ can be detected in the Li anode (Figure 5D) (Yan C. et al., 2016; Zhang et al., 2018). These results are the same as the XRD result in Figure 4k, which also further confirm the reaction as the Equations (2) and (3). Due to adding AgNO_3_ on the surface of the Li anode, we construct outstanding protecting layers with LiAg, LiN_3_, and LiN_x_O_y_. According to the result of the XRD and XPS spectrum, we propose that there is a difunctional protecting layer on the surface of the Li anode to suppress Li dendrites and protect it from being decomposed by electrolyte. This difunctional protecting layer contains the LiAg alloy and the modified SEI by LiNO_3_. And, it can be explained as the schematic of change on the AMLA anode during charge/discharge progress (Figures 4d,e). First of all, the LiAg alloy can suppress Li dendrites and maintain the integrity of the SEI film. The metal Ag has a great solubility in Li (9 at.% @ 145.5°C), which means that the metal Ag is lithiophilia. Moreover, the LiAg alloy is formed before the pure Li phase (Yan K. et al., 2016). Thus, the LiAg alloy particles are great nucleation sites of Li, because of the extreme lithiophilia. In addition, the metal Ag has the best conductivity in all of metal as the resistivity of 1.586 × 10^−8^ Ω·m at 25°C, which makes the LiAg alloy particles have better conduction than the pure Li (Zhang et al., 2018). To sum up, the distribution of the current and the nucleation of Li are uniform as the homogeneously distribution of LiAg nanoparticles. They result in even Li deposition. In addition, the Li diffusion in the phase LiAg alloy (>10^−8^ cm^2^ s^−1^; Ma et al., 2014) is far higher than in the metal Li (<10^−10^ cm^2^ s^−1^; Hiratani et al., 1988), which makes the Li deposit under the LiAg alloy. In a conclusion, the LiAg alloy will form firstly, and then the Li will deposit uniformly under the LiAg alloy (Yan K. et al., 2016), as shown in the Figure 4j. The thickness of deposited Li is about 5–10 μm. Hence, the LiAg alloy can suppress the Li dendrites and protect the integrity of SEI with the low expansion volumetric, which makes no violent voltage fluctuation during the cycles of the cell (Terlicka et al., 2019). Secondly, the modified SEI by LiNO_3_ can protect the Li from electrolyte. The LiNO_3_ made in the AMLA anode will react with electrolyte to form the Li_3_N and LiN_x_O_y_ (Shi et al., 2018). In addition, the great ionic conductivity of Li_3_N will make the Li^+^ diffuse through the SEI easily (Ma et al., 2014). Above, these two reacted productions will form dense and stable SEI with great ionic conductivity (Shen et al., 2018). Therefore, this SEI protects the Li from the electrolyte, which will prevent the reaction between the electrolyte and Li, thereby reducing electrolyte consumption (Shi et al., 2018). It also hinders the side reaction between the intermediate Li polysulfides (Li_2_S_x_, 4 ≤ x ≤ 8) and the Li anode to decrease the loss of active materials, when the AMLA anode is used in the Li-S battery (Figure 6a; Yan C. et al., 2016; Zhang K. et al., 2017).

The electrochemical performances of the Li-S cells are showed in the Figure 6b. These cells with different electrodes (the Li anode and AMLA) are tested by galvanostatic at 0.8375 mA cm^−2^ (0.5 C, 1 C = 1,675 mA g^−1^). As shown, the discharge capacity of Li-S cell with the AMLA anode remains as high as 666.7 mAh g^−1^ and the capacity retention after 500 cycles is 60.8%. However, the cell with the Li anode shows a capacity of 310.8 mAh g^−1^ with a capacity retention of only 29.1%, which is just half of the AMLA. What's more, the morphologies of the AMLA are still smooth without any Li dendrites the same as it in symmetric cells (Figures 6c–f). In a conclusion, the protecting layer formed by the AgNO_3_ can protect the Li electrode by suppressing the Li dendrites and prolong the cycle lifetime by hindering the corrosion reaction in the Li-S cell. This effect can be furtherly studied by the XPS in the Figures 6g,h. S 2p spectrum of the Li anode shows two main peaks at binding energy of 163.49 eV for Li_2_S and 164.48 eV for Li_2_S_2_, which are the reaction products of Li with intermediate Li polysulfides (Figure 6h). These two composites decrease the active materials in the S cathode. However, the rations of Li_2_S_2_ and Li_2_S are much lower than ${\text{SO}}_{3}^{2-}$ (168.11 eV), R-SO_2_-R′ (169.12 eV), and ${\text{SO}}_{4}^{2-}$ (170.37 eV), which are the compositions of the SEI film in the S 2p spectrum of the AMLA (Figure 6g). It means that there are negative Li_2_S_2_ and Li_2_S and the corrosion reaction is suppressed. The difunctional protecting layer formed in the AMLA is favorable to suppress the Li dendrites, and protect the Li from the corrosion reaction by the intermediate Li polysulfides at the same time.

## Conclusions

In general, we introduce an effective and simple strategy to improve the Li anode by forming the difunctional protecting layer on the Li anode. The difunctional protecting layer is manufactured through dropping the solution of AgNO_3_ on the Li anode directly. They suppress the Li dendrites by depositing Li^+^ under them uniformly and hinder the Li anode from the electrolyte to suppress the corrosion reaction. Comparing to the Li anode, the AMLA shows superior electrochemical performance with stable overpotential in the symmetric cell and longer cycling lifetime in Li-S battery. The cell with the AMLA can be cycled more than 50 h at 5 mA cm^−2^ with the steady overpotential of lower than 0.2 V. In the Li-S battery, after 500 cycles, the AMLA can still remain the high discharge specific capacity of 666.7 mAh g^−1^ as capacity retention rate of 60.8 % at 0.8375 mA cm^−2^ (0.5 C, 1 C = 1,675 mA g^−1^). We believe this simple approach can improve the Li anode to offer great guidance for further application of next-generation batteries, such as Li-S batteries.

## Data Availability

All datasets generated for this study are included in the manuscript and/or the supplementary material.

## Author Contributions

CS developed the concept. XL designed the experiments. JG conducted the experiments. ZF and JY built the cells. YZ, ZT, and LS carried out the performance characterizations. CS and KX co-supervised the research. JG and ZF co-wrote the manuscript. All authors discussed the results and commented on the manuscript.

### Conflict of Interest Statement

YZ, ZT, and LS were employed by company Shaanxi Coal and Chemical Technology Institute Co., Ltd. The remaining authors declare that the research was conducted in the absence of any commercial or financial relationships that could be construed as a potential conflict of interest.
